# Assessing the Antecedents and Consequence of Enterprise Transformation: A Quantitative Approach

**DOI:** 10.3389/fpsyg.2021.813858

**Published:** 2022-01-12

**Authors:** Haiyan Song, Tanaporn Hongsuchon, Santhaya Kittikowit, Zhe Dong

**Affiliations:** ^1^Trade and Economic Management School, Huzhou Vocational and Technical College, Huzhou, China; ^2^Chulalongkorn Business School, Chulalongkorn University, Bangkok, Thailand

**Keywords:** technological innovation, enterprise transformation, corporate performance, market pressure, multiple-group analysis

## Abstract

With the negative impact of COVID-19, the continuous recession of economic globalization, and the increasing market competition, enterprise transformation gradually becomes the theme of enterprise management. Although more and more scholars and companies have paid attention to the importance of enterprise transformation, most of the research on it is still at the qualitative level of theoretical descriptions and lacks a comprehensive consideration and empirical research on its motivation and performance. In view of this, this study analyzes the overall driving effect of technological innovation and the internal and external environment on enterprise transformation from the perspective of its drivers and analyzes in depth its causes and consequences for different industries (construction and real estate industries). The study also analyzes the antecedents and consequences of enterprise transformation and its differences in different industries (construction and real estate). In this study, a sample of middle and senior management of 10 companies with a valid sample of 401 is collected. Structural equation modeling results indicate that competitive advantage, technological innovation, and market pressure significantly affect enterprise transformation, which is an antecedent of corporate performance. Further, the results of the multiple-group analysis also reveal some significant differences between the theoretical models of the construction and real estate communities. Finally, suggestions are made based on the findings.

## Introduction

In recent years, scholars have proposed many research themes and frontier hotspots in the field of enterprise transformation research, including transformation motives (Yang et al., 2017; Zhao et al., 2020; Vrolijk, 2021), global value chain (Gereffi and Lee, 2016; Song et al., 2016; Reis et al., 2021), transformation strategies and paths (Zhu, 2019; Efogo, 2020; Yang et al., 2021), transformation models (Schaltegger et al., 2016; Li et al., 2019; Cao, 2020), transformation experiences and case studies (Mao and Wen, 2012; Zhou et al., 2018; Jia et al., 2021). Obviously, as a competitive strategy to enhance the competitiveness or high value-added of enterprises, enterprise transformation has become a hot topic of attention in academic and practical circles and has become a meaningful and rich topic of discussion and management research in the field of business-market relationship. In the context of COVID-19 and China’s economic transformation, it has become the mainstream of business management for companies to respond positively to the transformation (Yuan et al., 2020; Fu et al., 2021; Kong et al., 2021). Therefore, enterprise transformation has become increasingly important in market competition and relying on transformation to maintain continuous change and innovation capabilities extremely important for companies to survive and thrive in a rapidly changing business environment. The existing literature analyzes the importance of enterprise transformation theory from the following aspects.

First, enterprise transformation helps to alleviate the pressure of upgrading. The timely choice of transformation by firms helps firms avoid the pressure that the original industry has become difficult to provide sufficient growth space for firms (Crossa, 2021), helps improve the competitive position of firms within the industry, influences firms to enter industries with profitability (Leao and da Silva, 2021), helps firms to enter more profitable capital and technology-intensive fields (Poon, 2004), influences firms to move from low value-added activities to high value-added activities (Kong et al., 2021), and influences firms to move from simple activities to complex design and R&D innovation, thus improving competitiveness and business performance (Jia et al., 2021).

Second, enterprise transformation helps to enhance core competitiveness (Bell and Albu, 1999). Enterprise transformation propels enterprises to transform from OEM to ODM, or even advanced forms such as DMS and EMS, to enhance technological competitiveness; it propels enterprises to transform from OEM and ODM to OBM, to enhance brand competitiveness; or it propels enterprises to exploit technological synergies, thus entering industries with greater value-added potential (Zhu et al., 2006). By moving from contract manufacturing (OEM) to R&D and design (ODM) and establishing independent brands (OBM), improving product quality and enhancing international competitiveness (Mao et al., 2010), and ultimately achieving independent innovation and transformation of enterprises (Amsden, 1989; Zhao et al., 2020).

Third, enterprises transformation helps increase value-added. Enterprises actively engage in transformation activities can make enterprises restructure production factors, influence the improvement of input-output efficiency, and realize process upgrading; drive enterprises to introduce advanced production lines, influence the improvement of existing products and the launch of new products, and realize product upgrading; enable enterprises to increase the added value of products, influence the trade-off of existing functions and the acquisition of new functions, and realize function upgrading; then promote the transfer of industrial knowledge, which acts on related industries, and then realizes industrial upgrading (Gereffi and Lee, 2016). Therefore, for enterprises, “realizing independent innovation and transformation path” can not only “explore new business and development directions,” but also “obtain and maintain a stable competitive advantage” through the increase of added value in the industrial chain (Humphrey and Schmitz, 2002; Kaplinsky and Morris, 2003; Kong et al., 2021).

Thus, although the importance of enterprise transformation has been recognized by many enterprises and academics, and a series of valuable results have been obtained from related researches, the existing literature on the drivers of enterprise transformation still has several shortcomings in the following aspects. The existing literature focuses on the driving effect of independent variables on enterprise transformation, but there is no research on the driving mechanism of technological innovation under the combined effect of internal and external conditions. Enterprise transformation is influenced by the external environment, especially the competitive market pressure faced by technological innovation, and is also constrained by the competitive advantage in the internal environment. A comprehensive analysis of technological innovation and its internal and external influencing factors is beneficial for a deeper understanding of the driving effect of enterprise transformation. Studies have considered technological innovation as an important antecedent driver of enterprise transformation, ignoring the evaluation of the acquisition of sustainable competitiveness and the improvement of the added value of products and services, that is, the evaluation of the corporate performance of enterprise transformation. In addition, research has neglected the evaluation of the acquisition of sustainable competitiveness and the improvement of added value of products and services, that is, the measurement of corporate performance as a result of enterprise transformation. The existing studies mainly focus on samples from developed countries, and there is a lack of research on the construction of the theoretical system of enterprise transformation in China.

Based on the above discussion, this study takes COVID-19 as the research background, constructs a theoretical model of internal and external conditions affecting enterprise transformation, takes local real estate enterprises as the research object, and takes middle and senior managers of enterprises as effective samples, uses structural equation modeling to verify the explanatory power of the mechanism of action of enterprise transformation and upgrading, analyzes how technological innovation has a driving effect on enterprise transformation and upgrading under the overall market pressure and competitive advantage, so as to understand the important influencing factors affecting enterprise transformation and upgrading and their antecedents and consequences. This study provides practical guidance on the role mechanism of enterprise transformation under COVID-19 and theoretical support for improving the mechanism of enterprise transformation by deeply exploring its inherent theoretical logic and influence mechanism.

## Literature Review and Research Hypotheses

### The Driving Role of Technological Innovation

As an important driver of enterprise transformation, technological innovation is conducive to the continuous improvement of product or service quality, which helps enterprises to meet the consumer’s consumption intention and demand well, thus enhancing the market share and enterprise market competitiveness. In addition, technological innovation contributes to the renewal of production processes and procedures, the improvement of production efficiency and the reduction of production costs, and ultimately the improvement of business performance (Hame, 1998; Cohen, 2010). Corporate performance is an important evaluation indicator of business operation and development. Its financial indicators can reflect the productive capacity of the company, the synergistic effect of the division of labor and cooperation among the team members of the company, and the trust and sense of belonging of the employees to the development of the company (Hackman, 1987).

It has been shown that technological innovation can enhance corporate performance (Jefferson et al., 2006). Xu et al. (2018) took equipment manufacturing enterprises as the research object and argued that corporate technological innovation had a significant positive impact on corporate performance from the perspective of low-carbon theory through a combination of qualitative and quantitative methods. Based on the theory of disruptive innovation, Wu et al. (2013) showed through an empirical test of 201 firms’ data that technological innovation not only had a significant impact on corporate performance, but also found that the relationship between different combinations of technological innovation and market orientation have distinct effects on corporate performance.

Based on this, we propose the following hypothesis.

H1: Technological innovation is positively related to corporate performance.

Technological innovation has a driving role in enterprise transformation. To meet the continuous challenges of transformation, companies are required to improve their existing products, technologies, and market services through technological innovation in order to optimize their organizational structure, reduce production costs and increase operational efficiency (Zhang and Zhao, 2015). By relying on new technology development, new business development, optimization of business operation model and internal organizational restructuring, and even equipment renewal, enterprise transformation enters into higher value-added industries, which drives the improvement of production processes, manufacturing methods, and product quality, thus promoting enterprises from the lower end of the value chain to the higher end of the value chain and ultimately improve their competitiveness. Enterprise transformation can increase the share of revenue from new types of business, improve product quality and brand image, and increase the added value of products. Thus, technological innovation helps to drive transformation by changing the technological trajectory of the firm (Lin et al., 2015).

The high correlation that exists between technological innovation and transformation has been argued by prior studies. For example, in studying the factors influencing enterprise transformation in manufacturing industries of China, Kong (2012) examined the link between enterprise innovation behavior and enterprise size and enterprise transformation through statistical empirical evidence, and based on this, he used a binary choice model to find that enterprise innovation capability is the most critical factor of enterprise transformation. Bi et al. (2017) found that low-carbon technological innovation had a significant driving effect on manufacturing industry upgrading when they investigated the relationship between manufacturing industry upgrading and low-carbon technological breakthrough innovation. Based on the above discussion, we propose the following hypothesis.

H2: Technological innovation is positively related to enterprise transformation.

Technological innovation enables a company to provide more attractive products or services than its competitors, and to gain a sustainable competitive advantage in a highly competitive industry. Compared with competitors, technological innovation enables companies to gain economies of scale and learning curve effects in the market competition, which helps companies to provide products or services that are acceptable to customers at the lowest production cost and keep the cost of products or services at the leading level in the industry. Equally important, technological innovation is used by firms to provide unique products or services to customers in certain segments of the value chain and win their favor. As an important source of low-cost competitive advantage or differentiated competitive advantage, technological innovation has a great impact on the competitive advantage of a company.

It has been shown that technological innovation has a significant role in determining the relative cost position or differentiation of products. Mang (1998) pointed out that by investing more in R&D and implementing product differentiation strategies, one can win a dominant position in a competitive market and gain competitive advantage. Wang (2007) concluded through an empirical study that indigenous technological innovation is a significant influencing factor to improve the competitive advantage of manufacturing trade, and further drive technological innovation. Thus, this study suggests that there is a significant linkage between technological innovation and competitive advantage. Based on this, we propose the following hypothesis.

H3: Technological innovation is positively related to competitive advantage.

### The Mechanism of Enterprise Transformation on Corporate Performance

Enterprise transformation helps enterprises to enhance their competitive advantage and obtain great economic benefits. Through transformation, enterprises can participate in social division of labor in the capital and technology-intensive economy at the higher end of the value chain (Gereffi, 1999), enhance their competitive advantage and produce value-added products with technological and market advantages (Humphrey and Schmitz, 2004), and shift from the role of producers of labor-intensive low-value products to manufacturers of capital- or technology-intensive products (Poon, 2004), thus gaining competitive advantage and high returns. At the same time, in order to cope with the crisis caused by the change of business environment or the challenge caused by the renewal of the industry in which the company is located (Porter, 1991), it is also a strategic motive for companies to obtain core competitiveness and long-term development to break through the bottleneck of development and find the direction of transformation and greater profitability through transformation (Blumenthal and Haspeslagh, 1994). Therefore, enterprise transformation can enable companies to obtain great corporate performance.

The existing literature has pointed out the relationship between enterprise transformation and corporate performance. Kaplinsky and Morris (2003) distinguished and explained the relationship between enterprise transformation and corporate performance resulting from transformation. By distinguishing the types of transformation, Qiu and Liu (2015) pointed out that product upgrading could directly contribute to corporate performance or indirectly affect the increase of corporate performance by promoting technological innovation; while functional upgrading had a positive impact on corporate performance only through technological innovation. The positive impact of functional upgrading on corporate performance can only occur through technological innovation, and functional upgrading without technological innovation will have a negative impact on corporate performance. As a result, we propose the hypothesis that.

H4: Enterprise transformation is positively related to corporate performance.

### The Driving Role of Market Pressure

In the competitive modern market, enterprises integrate internal conditions and external environmental resources and need to balance the interests of stakeholders’ needs in order to seek long-term survival and development.

Therefore, enterprises bring a huge market pressure for meeting the stakeholder’s interest needs, which forces them to actively respond to innovation and pay attention to scientific and technological issues in the market competition. By further research, Eiadat et al. (2008) argued that technological innovation not only positively affected the growth of corporate performance and the winning of competitive advantage, but also responded to pressures from the market side (including consumers, suppliers, and peer competitors). Jennings (1995) stated that demand pressure from stakeholders, both internal and external to the firm, would force firms to focus on innovation issues and take the initiative to engage in technological innovation. Zhai and Bi (2016) pointed out through a study of listed high-tech enterprises that when firms faced market pressure, the driving force of exploratory innovation investment and developmental innovation investment would be correspondingly increased through meta-innovation investment, with developmental innovation investment playing a much obvious role. From the perspective of asset specificity, Xu et al. (2015) found that although there was a significant difference in market pressure faced by different firms, as the investment in research and development increased, the firm would win the competitive advantage and excess profit.

In view of this, the following hypotheses are proposed in this study:

H5: Market pressure is positively related to technological innovation.

H6: Market pressure is positively related to corporate performance.

In addition, the continued intensification of competition will also have technology or products that are difficult to create long-term revenue for the company, which will lead to the further expansion of the market pressure faced by the company. As the pressure continues to increase, firms, in order to gain great revenue and sustained competitive advantage, will then make enterprise transformation or upgrade through technological innovation, thus successfully avoid market pressure (Li et al., 2005). Under the pressure of market competition and market pressure, enterprises will make fundamental transformation and changes to their business direction, strategic structure, and resource allocation, in an attempt to alleviate competition and market pressure, enhance social value and regain competitive advantage.

Many scholars have conducted research on this subject, expecting to help companies to survive the crisis and transform successfully. Clemons and Hann (1999) clearly argued that regular transformation of most firms had become increasingly important in the current competitive market. Mao and Wang (2006) pointed out through a study of local enterprises that enterprise transformation was a unique phenomenon in emerging economies because enterprises in emerging economies were facing greater market pressure than those in developed countries, and the study also pointed out that intense market pressures are also opportunities and spaces for corporate transformation and upgrading. Zhou and Yang (2013) took Guangdong foreign trade enterprises as the research object and found through structural equation modeling that the market pressure of SMEs and large enterprises had significant influence on the aspects of enterprise transformation and government policy support, but their current difficulties, transformation direction, and policy needs and other pressures had significant differences, so the government needed to introduce appropriate support policies according to the specific situation of different types of enterprises.

Based on this, we propose the following hypothesis.

H7: Market pressure is positively related to enterprise transformation.

### The Driving Role of Competitive Advantage

Resource-based theory suggests that the optimal combination and reallocation of a firm’s valuable, scarce, and inimitable resources is an important source of gaining competitive advantage (Barney et al., 2001). The key resources and key capabilities that companies have are the foundation for enterprise transformation and the starting point and beginning of enterprise transformation (Makadok, 2001; Makadok and Barney, 2001). Therefore, full understanding and judgment of key resources and capabilities is an important part of enterprise competitive advantage identification. As the competitive advantage of enterprise transformation, abundant key resources and capabilities become the antecedent determinants of enterprise transformation, which is an important prerequisite for enterprises to carry out transformation. On the one hand, capital accumulation, as a key resource, is conducive to the success of enterprise transformation by improving the level of technological innovation, enhancing product brand image, and establishing independent brands through good corporate performance (Forbes and Wield, 2002). On the other hand, the impact of different types of resources on corporate performance varies. The impact of resources underlying strategic alliances on corporate performance occurs under uncertain conditions of technological innovation or when the level of product innovation is low, and competitive advantage has a significant impact on market performance (Murray and Greenes, 2006). Strategic alliance partner resources act on the choice between partners and influence the adaptation between partners through partner choice, which in turn synergistically affects the performance of alliance business strategies (Luo, 1997). Remodeling the organizational structure and matching corporate resources facilitate the speed of transformation, capturing key factors of enterprise transformation such as management culture and innovation culture, reducing strategic restructuring and generating benefits, improving corporate performance, and gaining competitive advantage (Luo and Chi, 2005).

Competitive advantage can continuously create added value for customers, win customer satisfaction and loyalty, and obtain superior corporate performance over competitors. Protogerou et al. (2011) argued that competitive advantage could continuously create superior value for customers and enable firms to achieve superior performance, and further pointed out that competitive advantage led to performance including not only financial performance but also non-financial performance, such as product quality, customer satisfaction, etc. Wang (2016) showing that corporate core competencies had a positive effect on corporate performance. Through a questionnaire survey of 296 firms in China, and the results of the analysis with structural equation modeling and partial least squares, they found that corporate core competencies were a mediating variable of information technology affecting corporate performance. Therefore, we propose the following hypotheses.

H8: Competitive advantage is positively related to enterprise transformation.

H9: Competitive advantage is positively related to corporate performance.

**Figure 1:**
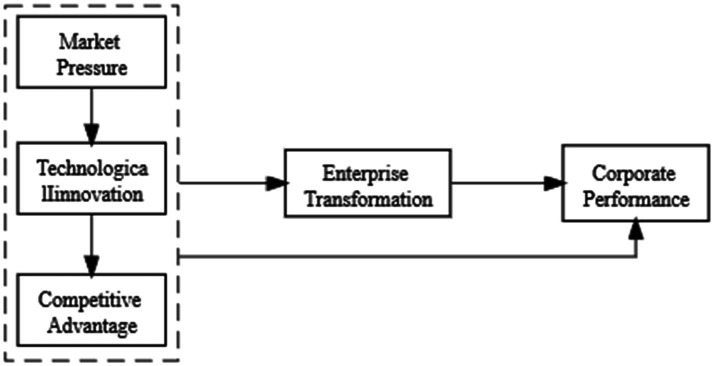
Theoretical model.

In summary, this study proposes a research model as shown in Figure 1.

## Materials and Methods

### Participants and Procedure

In this study, real estate companies and construction companies are selected as the research samples. The reasons are as follows, First, because the construction industry is currently facing the bottleneck of rising energy efficiency in buildings, and the in-depth promotion of transformation has become an important way for it to break through the bottleneck and achieve green and high-quality development. Second, due to the significant structural changes in the real estate market and the adjustment of relevant national policies, transformation has become a new development direction being explored by the real estate industry. Therefore, it is of more practical value to study the industry and enterprises where transformation is imperative. The questionnaires of this study are targeted at middle and senior managers of enterprises. The data collection of this study is completed through two rounds of questionnaire survey.

The first round of research, which started at the end of March and lasted until the end of June 2021, selected five real estate enterprises, and a total of 230 middle and senior managers in the real estate industry participated in the questionnaire survey, and a total of 208 valid questionnaires were obtained. The second round of research, which started from the beginning of July and lasted until the end of August 2021, selected five construction enterprises, and a total of 220 middle and senior managers of the construction industry participated in the questionnaire survey, and a total of 193 valid questionnaires were obtained. A total of 401 valid samples were obtained in the two rounds of research. Among them, 75.56% was male, 89.56% under 45 years old, 2.89% over 60 years old, 13.78% of master’s degree and above, 52.22% of bachelor’s degree, and 34% of specialist and below. Middle-level managers accounted for 66% and senior managers accounted for 34%; in terms of years of work in the positions held, 44.23% was below 5 years, 30.66% above 5 years and within 10 years, and 25.11% above 10 years.

### Measures

First, to ensure the reliability and validity of the measurement scales, the measurement questions in this study are all based on existing established scales, which are organized according to the research objectives through literature analysis. Among them, the scales from SSCI journals are translated and validated according to the recommendations of the back-translation method to ensure the quality of the questionnaire and its applicability in Chinese context. Second, to ensure the expert validity of the measurement questionnaire, five scholars in the field of transformation research and three corporate executives engaged in enterprise transformation are invited to review the questionnaire, which is finally formed through the joint suggestions from the academic and corporate practice communities. Third, the measurement questionnaire of this study consists of 23 items, and the measurement items are all measured using a Likert 7-point scale. The variables are measured as follows.

- Technological innovation: this study refers to the technological innovation research scale of Nietoa and Quevedo (2005), Xu et al. (2018), and Wang (2016), which consists of five questions, such as “the proportion of the company’s investment in technological innovation equipment is increasing” and “the proportion of the company’s technological innovation personnel to all R&D personnel is gradually increasing.”
- Corporate performance: this paper draws on the studies of Antoncic et al. (2007) and Jaakko et al. (2010). The scale consists of four questions, such as “the growth rate of the company’s market share is increasing year by year” and “the overall market competitiveness of the company’s products is higher than that of its main competitors in the same industry.”
- Enterprise transformation: In this paper, drawing on the studies of Jaworski and Kohl (1993), Li et al. (2008), and Jia and Zhao (2014), the scale consists of five questions, with sample questions such as “This enterprise can grasp the time of transformation in a timely manner,” “This enterprise has invested relative financial resources in transformation.” The scale consists of five items, such as “The company can grasp the transformation time in time” and “The company has invested relative resources in the transformation.”
- Market pressure: this article draws on the studies of Tang and Tang (2012), Wang et al. (2010a), and Cao and Chen (2017), with four questions. The sample questions are “Most of our customers have higher and higher demands on product quality” and “Most of our customers are very concerned about the development of new products in our company.”
- Competitive advantage: in this study, the scale consists of five questions, as studied by Dong et al. (2011) and Ma et al. (2014). Sample questions include “Compared with competitors, this company can provide products or services to customers at a lower cost,” “Compared with competitors, this company can provide multifunctional and high-performance products or services to customers,” etc.
- Control variables: in this study, the subjects’ gender, age, education, and years of job tenure are selected as control variables.

## Data Analysis

### Common Method Variance

Common method variance (CMV) is an overestimation of inter-construal correlations due to self-reported scales and is also influenced by the same measurement method, which reduces the variance between different constructs (Xu, 2015). Thus, CMV is the error of the measurement instrument and measurement error affects the validity of the conclusions of measuring the relationship between constructs (Podsakoff et al., 2003).

CMV *post hoc* detection can be handled by applying SEM, where different conformational variables are analyzed using a single CFA model, where a good correlation between conformations means that there is CMV and CFA yields a good fitness (Korsgaard and Roberson, 1995; Mossholder et al., 1998). Single CFA assessed the increase in CMV to CMV nested competition pattern, its complexity and assessed whether the increase in cardinality is significant (Podsakoff and Organ, 1986; McFarland and Sweeny, 1992). Single CFA analysis shows that *χ*^2^ = 2130.080 and DF = 230, multi-factor CFA analysis shows that *χ*^2^ = 563.311 and DF = 220, two models ΔDF = 230–220 = 10 and Δ*χ*^2^ = 2130.080–563.311 = 1566.769, and their significance is calculated by applying distcale software. The significant difference *p* < 0.00, indicates that the null hypothesis is rejected, so the two models are not different and no common method deviation exists between the conformational surfaces, and the results are shown in Table 1.

**Table 1:** Comparison of single-factor and multi-factor model.

Model	Single-factor CFA	Multi-factor CFA
DF	230	220
△DF		10
*χ* ^2^	2130.080	563.311
△*χ*^2^		1566.769

### Confirmatory Factor Analysis

CFA is a part of SEM analysis. The variable reduction of CFA measurement model in this study is based on Zhang et al. (2021) two-stage model modification (Anderson and Gerbing, 1998). The measurement model must be tested before performing the structural model evaluation. A complete SEM model report can only be carried out if the measurement model is reasonably acceptable (Kline, 2005).

In this study, CFA analysis is performed on all dimensions, and the results are shown in Table 2. The standardized factor loadings of all dimensions are between 0.693 and 0.862, the composite reliability is between 0.843 and 0.912, and convergence validity is between 0.575 and 0.675, meeting all the standards of Fornell and Lacker (1981) with standardized factor loadings greater than 0.50, composite reliability greater than 0.60, and convergence validity greater than 0.50 (Verbeke et al., 2014; Hair et al., 2017). Therefore, the model meets the standard, and all aspects have good convergence validity.

**Table 2:** Confirmatory factor analysis.

Construct	Item	Significance of Estimated Parameters			Item Reliability			Construct Reliability	Convergence Validity
		*Unstd.*	*SE*	*value of z*	*value of p*	*Std.*	*SMC*	*CR*	*AVE*
TI	TI1	1.000				0.719	0.517	0.843	0.575
	TI2	1.064	0.069	15.346	^***^	0.797	0.635		
	TI3	1.106	0.071	15.512	^***^	0.807	0.651		
	TI4	0.898	0.066	13.671	^***^	0.704	0.496		
MP	MP1	1.000				0.740	0.548	0.891	0.621
	MP2	1.068	0.064	16.812	^***^	0.815	0.664		
	MP3	1.052	0.064	16.549	^***^	0.802	0.643		
	MP4	1.012	0.063	16.109	^***^	0.782	0.612		
	MP5	1.033	0.063	16.467	^***^	0.798	0.637		
CA	CA1	1.000				0.790	0.624	0.912	0.675
	CA2	1.064	0.053	20.129	^***^	0.862	0.743		
	CA3	1.011	0.055	18.412	^***^	0.804	0.646		
	CA4	1.021	0.054	18.993	^***^	0.823	0.677		
	CA5	1.025	0.054	19.116	^***^	0.828	0.686		
ET	ET1	1.000				0.699	0.489	0.883	0.602
	ET2	1.057	0.071	14.961	^***^	0.775	0.601		
	ET3	1.079	0.070	15.374	^***^	0.799	0.638		
	TET4	1.120	0.072	15.659	^***^	0.815	0.664		
	ET5	1.062	0.070	15.157	^***^	0.786	0.618		
CP	CP1	1.000				0.693	0.480	0.843	0.575
	CP2	1.071	0.073	14.696	^***^	0.787	0.619		
	CP3	1.111	0.075	14.782	^***^	0.792	0.627		
	CP4	1.003	0.071	14.188	^***^	0.756	0.572		

STD, Standardized Factor Loadings; SMC, Square Multiple Correlations; CR, Composite Reliability; AVE, Average Variance Extracted; TI, Technological Innovation; MP, Market Pressure; CA, Competitive Advantage; ET, Enterprise Transformation; CP, Corporate Performance.

*** *p* < 0.001.

### Discriminant Validity

The discriminant validity analysis is to examine whether two different variables in the statistics are different or not. In this study, the AVE method is used to evaluate the discriminative validity. Fornell and Lacker (1981) propose the square root of the AVE with the correlation between the construct and other constructs in the model, which means that the variables have discriminative validity. As shown in Table 3, the square roots of the AVE on the diagonal are larger than the correlations between constructs, indicating acceptable discriminant validity. Therefore, this study has good discriminative validity.

**Table 3:** Discriminant validity.

	AVE	MP	TI	CA	ET	CP
MP	0.621	**0.788**				
TI	0.575	0.560	**0.758**			
CA	0.675	0.344	0.614	**0.822**		
ET	0.602	0.539	0.685	0.636	**0.776**	
CP	0.575	0.689	0.708	0.600	0.703	**0.758**

The items on the diagonal on bold represent the square roots of the AVE. Off-diagonal elements are the correlation estimates. TI, Technological Innovation; MP, Market Pressure; CA, Competitive Advantage; ET, Enterprise Transformation; CP, Corporate Performance.

### Model Fit Degree

The structural model analysis is performed by the method of great likelihood estimation, and the analysis results include model fit, significance test of the research hypotheses, and interpretable variance (*R*^2^). The research hypothesis of SEM is sample covariance matrix = model covariance matrix. However, SEM is a large sample analysis method, so the value of *p* is very easy to be less than 0.05 and often wrongly reject the hypothesis and get the conclusion that the model is not good, so Kline (2011) and Schumacker and Lomax (2010) suggested that the degree of model fit should not be determined by the value of *p*, but to report a variety of different fit indicators to determine whether the model fit is good.

The fit metrics in this study apply the 194 international academic journal (SSCI) papers explored in the study of Jackson et al. (2009) as a blueprint for applying model fit analysis and report the results of this study with nine most widely used fit metrics. These include ML*χ*^2^, DF, Normed Chi-sqr (*χ*^2^/DF), RMSEA, SRMR, TLI (NNFI), CFI, GFI, and AGFI. The results of the Bollen-Stine Bootstrap corrected model fit, and the Bollen-Stine Bootstrap corrected model fit are shown in Table 4. After that, all the fit metrics of this study have passed, showing that the results of this study are acceptable models.

**Table 4:** Model fit criteria and test results.

Model fit	Criteria	Model fit of research model
ML*χ*^2^	The small the better	467.594
DF	The large the better	221
Normed Chi-sqr (*χ*^2^/DF)	1 < *χ*^2^/DF < 3	2.12
RMSEA	<0.08	0.05
SRMR	<0.08	0.07
TLI (NNFI)	>0.9	0.96
CFI	>0.9	0.96
GFI	>0.9	0.93
AGFI	>0.9	0.91

### Regression Coefficient

In this research model (as shown in Table 5), market pressure (MP; *b* = 0.581, *p* < 0.001) significantly affects technological innovation (TI), technological innovation (TI; *b* = 0.665, *p* < 0.001) significantly affects competitive advantage (CA). Competitive advantage (CA; *b* = 0.281, *p* < 0.001), technological innovation (TI; *b* = 0.304, *p* < 0.001) and market pressure (MP; *b* = 0.207, *p* < 0.001) significantly affect enterprise transformation (ET), technological innovation (TI; *b* = 0.206, *p* < 0.001), market pressure (MP; *b* = 0.352, *p* < 0.001), competitive advantage (CA; *b* = 0.156, *p* < 0.001) and enterprise transformation (ET; *b* = 0.231, *p* < 0.001) significantly affect corporate performance (CP). Therefore, all hypotheses are established.

**Table 5:** Regression coefficient.

DV	IV	Unstd	*SE*	Unstd./*SE*	*value of p*	Std.	*R* ^2^
TI	MP	0.581	0.062	9.433	^***^	0.56	0.314
CA	TI	0.665	0.063	10.538	^***^	0.614	0.377
ET	CA	0.281	0.046	6.162	^***^	0.346	0.578
	TI	0.304	0.059	5.114	^***^	0.345	
	MP	0.207	0.047	4.375	^***^	0.226	
CP	TI	0.206	0.06	3.43	^***^	0.228	0.691
	MP	0.352	0.051	6.868	^***^	0.375	
	CA	0.156	0.046	3.42	^***^	0.187	
	ET	0.231	0.067	3.478	^***^	0.225	

*** *p* < 0.001.

TI, Technological Innovation; MP, Market Pressure; CA, Competitive Advantage; ET, Enterprise Transformation; CP, Corporate Performance.

### Multiple-Group Analysis

In this study, we want to understand whether there is a significant difference between the effects of the two industries of construction and real estate in the model constructs, so we use the comparison of clusters in the structural equation model (Deng et al., 2008; Abram et al., 2017) and analyze the results of the estimation of each of the two groups of people in the structural equation model in the construction industry and real estate industry respectively, followed by setting the regression coefficients of the two groups to be equal, and if the value of *p* of the check results is less than 0.05, indicating that the two slopes are significantly different, and vice versa (Pousette and Hanse, 2002; Smith et al., 2016). The analysis is organized into Table 6, there are nine regression coefficients in the model, and the results of the analysis of the construction and real estate industries are shown in the table, and the comparison result *Z* > 1.96 indicates that there is a significant difference between the regression coefficients of the two groups in the construction and real estate industries. It can be seen that there are five groups with significant differences in the study results, which are the effect of competitive advantage on enterprise transformation, the effect of technological innovation on enterprise transformation, the effect of technological innovation on corporate performance, and the effect of technological innovation on corporate performance, technological innovation on corporate performance, market pressure on corporate performance, and competitive advantage on corporate performance. The rest of the paths are not significant.

**Table 6:** Multiple-group comparison regression coefficients.

DV	IV	Construction		Real estate		Regression weight comparison		
		Estimate	*SE*	Estimate	*SE*	diff.	*value of z*	*value of p*
TI	MP	0.539	0.079	0.699	0.121	−0.16	1.106	0.27
CA	TI	0.903	0.119	0.696	0.091	0.207	1.384	0.167
ET	CA	0.203	0.052	0.806	0.124	−0.603	4.5	0
	TI	0.499	0.105	−0.1	0.1	0.599	4.133	0
	MP	0.087	0.056	0.273	0.08	−0.186	1.889	0.06
CP	TI	0.397	0.125	0.026	0.126	0.371	2.087	0.038
	MP	0.417	0.07	−0.059	0.116	0.476	3.499	0.001
	CA	−0.042	0.056	1.046	0.29	−1.088	3.679	0
	ET	0.212	0.119	−0.029	0.26	0.241	0.84	0.402

TI, Technological Innovation; MP, Market Pressure; CA, Competitive Advantage; ET, Enterprise Transformation; CP, Corporate Performance.

## Results

### Research Conclusion

This study examines the drivers of local enterprise transformation in China and its internal and external influencing mechanisms by taking middle and senior managers in the real estate and construction industries as the research subjects. Based on the results of the empirical analysis of structural equation modeling, this study provides a more complete explanation of the mechanisms and influencing factors of the local enterprise transformation in China under COVID-19.

First, Validated the role of internal and external factors in influencing firm transformation. This study shows that technological innovation and its internal and external environment have significant theoretical explanatory power for China’s enterprise transformation. Although theoretical studies on enterprise transformation in developed countries are abundant and have become a hot topic of social concern, research on enterprise transformation in China, especially empirical studies, is extremely limited. In view of the differences in enterprise management in terms of market operating environment, management philosophy, and business practices, it is necessary to examine the drivers of enterprise transformation and its performance in China from a theoretical perspective. Based on the theoretical foundation and literature review, this study examines the effects of market pressure, technological innovation, and competitive advantage on enterprise transformation in the Chinese context. Overall, market pressure, technological innovation, and competitive advantage are important antecedent influences on enterprise transformation and have positive effect on enterprise transformation. Among them, technological innovation is a very important predictor and main path of enterprise transformation. It is also found that technological innovation positively influences the competitive advantage of the firm and is also positively influenced by market pressure, which together affects the transformation. In addition, market pressure (MP; *b* = 0.207, *p* < 0.001) also plays a positive role in enterprise transformation. Meanwhile, the positive driving effect of technological innovation on enterprise transformation is more significant than that of competitive advantage and market pressure. Internal and external antecedents have a significant positive effect on corporate performance.

Second, Validated the mechanism of the impact of enterprise transformation on corporate performance. This study shows that corporate transformation has a significant effect on corporate performance, however, the driving effect of enterprise transformation motivation and its performance overall consideration varies significantly. On the one hand, there are differences in the driving effects of antecedent influences of enterprise transformation. The structural equation modeling analysis revealed that competitive advantage (*b* = 0.281, *p* < 0.001), technological innovation (*b* = 0.304, *p* < 0.001) and market pressure (*b* = 0.207, *p* < 0.001) had significant effects on enterprise transformation (ET). The results show that competitive advantage (CA; *b* = 0.281, *p* < 0.001) is a significant external antecedent influence on enterprise transformation and has a significant contribution to enterprise transformation. On the other hand, the path analysis through structural equation modeling shows that there are differences in the driving effects of the antecedent influences of corporate performance. Specifically, the situation is as follows. First, market pressure (MP; *b* = 0.352, *p* < 0.001), and competitive advantage (CA; *b* = 0.156, *p* < 0.001) are important external influences on corporate performance, but market pressure has a greater impact on corporate performance than competitive advantage and is the main external source of corporate performance growth. Second, enterprise transformation (ET; *b* = 0.231, *p* < 0.001), technological innovation (TI; *b* = 0.206, *p* < 0.001) are important internal influences on corporate performance, but enterprise transformation has a greater positive effect on corporate performance than technological innovation and is an important internal source of corporate performance growth. Internal and external antecedents have a significant positive effect on corporate performance. Internal and external important antecedents jointly drive the growth of corporate performance.

Third, revealed the significant differences of transformation mechanism between real estate and construction industries in terms of transformation. The multiple-group analysis reveals that the overall mechanism of action based on technological innovation has different effects on real estate and construction industries. Specifically, there are five groups of regression coefficients with significant differences between real estate and construction firms, namely, the driving effect of competitive advantage on transformation, the driving effect of technological innovation on enterprise transformation, the driving effect of technological innovation on corporate performance, market pressure on corporate performance, and competitive advantage on corporate performance; the remaining four groups, namely, the effect of market pressure on technological innovation, the effect of technological innovation on competitive advantage, the effect of market pressure on transformation, and the effect of enterprise transformation on corporate performance. The effect of transformation on corporate performance is not significant. This shows that there are significant differences in the effects of market pressure, technological innovation, and competitive advantage on corporate performance. Among them, the significance of competitive advantage on corporate performance is significantly stronger in the real estate industry than in the construction industry, while the effects of technological innovation and market pressure on corporate performance are stronger in the construction industry than those in the real estate industry. The effects of technological innovation and market pressure on corporate performance in the construction industry are stronger than those in the real estate industry. Meanwhile, there are significant differences in the effects of competitive advantage and technological innovation on enterprise transformation. Among them, the driving effect of technological innovation on enterprise transformation is stronger in construction than that in real estate industry, while the driving effect of competitive advantage on enterprise transformation is stronger in real estate industry than that in construction industry.

### Theoretical Contributions

First, it promotes the overall study of the antecedents and consequences of the mechanism of enterprise transformation. This study analyzes the core factors driving enterprise transformation from the perspective of enterprise transformation drivers, and proposes the overall influence of market pressure, technological innovation and competitive advantage on enterprise transformation, while technological innovation is driven by market pressure and transformed into competitive advantage. In particular, technological innovation is considered as an important antecedent driver of enterprise transformation under the constraints of market pressure and driven by competitive advantage, and the outcome of enterprise transformation, that is, corporate performance, is considered. The findings of this study help to better understand the root causes of enterprise transformation and evaluate the results and provide guidance for companies to further understand and transform their business practices to improve corporate performance and market competitiveness.

Second, it promotes the contribution to enterprise transformation theory. This study examines the mechanism of transformation through empirical research from a holistic perspective of enterprise transformation motivation and performance. Whether enterprises can make full use of technological innovation and turn it into opportunities and drivers of enterprise transformation requires the overall driving force of market pressure, technological innovation, and competitive advantage. When making transformation decisions, enterprises should consider the positive impact of technological innovation, market pressure on technological innovation, and the competitive advantage brought by technological innovation. In order to realize the synergistic effect of the overall driving factors and to lay a solid foundation for enterprise transformation to achieve superior performance, we should consider the three aspects as a whole.

### Practical Implications

The issue of China’s local enterprise transformation needs urgent attention. The details are as follows.

First, the most important thing is that, with the transformation of China’s economic structure and the increasingly difficult environment for enterprises to survive, more and more enterprises are seeking survival and development through transformation, and the number of enterprises undergoing transformation is bound to increase. Although China’s enterprise transformation space is huge, the causes and consequences of transformation have not received due attention, if not timely enterprise transformation of the important antecedent factors and their driving effect of all-round, multi-level research, it is very likely to ignore the pressure of transformation, and thus produce the negative impact of transformation, which is the failure of enterprises aspiring to obtain excellent performance through transformation practice. In addition, enterprise managers should pay attention to the role of internal and external factors on enterprise transformation. This study finds that technological innovation, actively driven by market pressure, has the greatest effect on enterprise transformation, and is also the greatest factor affecting corporate performance. Since technological innovation is the heavy source driving enterprise transformation, companies should break through the bottlenecks affecting technological innovation, enhance their core competitiveness by developing core technologies, and promote enterprise. More importantly, the enterprise transformation in different industries is the most important factor in the value chain.

Second, there are significant differences in the driving effect of enterprise transformation in different industries. We should not simply base on the driving effect of enterprise transformation, but should guide the practice of enterprise transformation for different industries by combining their own industry characteristics and market conditions.

For the construction industry, the positive impact of technological innovation on enterprise transformation and corporate performance is more significant, and enterprise transformation should take advantage of it. We should focus on using technological innovation to drive enterprise transformation. In other words, the success of corporate transformation is caused by increasing the investment in technological innovation, thus reducing the pressure of corporate transformation and obtaining greater corporate performance. At the same time, the positive effect of market pressure on corporate performance should be properly utilized, that is, the market pressure faced by the company should be turned into the motivation for corporate transformation, which will lead to the growth of corporate performance instead of the decline of performance.

For the real estate industry, the driving effect of competitive advantage on enterprise transformation and corporate performance is more significant than that of the construction industry, and enterprise transformation should take advantage of this. Therefore, enterprises in the real estate industry should pay more attention to the positive influence of competitive advantage while giving full play to the advantages of technological innovation and transforming the disadvantages of market pressure. In the increasingly competitive real estate industry under the market economy, companies with competitive advantage often become the winners. This is because when a company undergoes transformation, the more prominent competitive advantage can help the company reduce the pressure of upgrading and gain the ability to cope with environmental challenges. If a real estate company implements a competitive strategy that is difficult or too costly for its competitors to imitate, it gains a sustainable competitive advantage. For example, Wanda Commercial Real Estate’s business model innovation based on the smile curve theory is representative of a company that gains long-term competitive advantage and earns above-average profits. A real estate company with a competitive advantage can win the market and achieve above-average profits with good growth prospects, while a company without a competitive advantage will be outperformed by its competitors and suffer from mediocre corporate performance.

### Research Limitations and Future Research Directions

Although this study strictly follows the requirements of the questionnaire and empirical research to conduct multi-group analysis, the research perspective and ideas have certain advantages over existing studies and have practical significance for enterprise transformation activities, there are still some limitations for improvement. First, there are limitations in the sample source of this study. The questionnaire survey of middle and senior managers around “enterprise transformation” is in itself a complex and difficult topic. However, future research can expand the scope of data collection and conduct structural equation modeling with larger samples to enhance the generalizability of the research results.

Second, there are limitations in the sample sources of clusters in this study. Although this study is an advanced paradigm of cluster comparison analysis, which has significant superiority over general empirical studies, the cohort of this study is limited to the construction and real estate industries only, and future studies can collect data in a wider range of industries, such as small and medium-sized enterprises, private technology-based enterprises, manufacturing and other representative industries of transformation, and conduct hierarchical linear modeling studies in a larger industry cluster to enhance the generalizability of the study results.

## Data Availability Statement

The raw data supporting the conclusions of this article will be made available by the authors, without undue reservation.

## Ethics Statement

Ethical review and approval was not required for the study on human participants in accordance with the local legislation and institutional requirements. Informed consent was obtained from all subjects involved in the study.

## Author Contributions

HS and TH: conceptualization. HS and ZD: data curation, formal analysis, and investigation. HS, TH, and SK: writing original draft and writing – review and editing. All authors have read and agreed to the published version of the manuscript.

## Conflict of Interest

The authors declare that the research was conducted in the absence of any commercial or financial relationships that could be construed as a potential conflict of interest.

## Publisher’s Note

All claims expressed in this article are solely those of the authors and do not necessarily represent those of their affiliated organizations, or those of the publisher, the editors and the reviewers. Any product that may be evaluated in this article, or claim that may be made by its manufacturer, is not guaranteed or endorsed by the publisher.
